# Electrically Tuneable Optical Diffraction Gratings Based on a Polymer Scaffold Filled with a Nematic Liquid Crystal

**DOI:** 10.3390/polym13142292

**Published:** 2021-07-13

**Authors:** Dejan Bošnjaković, Mathias Fleisch, Xinzheng Zhang, Irena Drevenšek-Olenik

**Affiliations:** 1 Faculty of Mathematics and Physics, University of Ljubljana, Jadranska 19, 1000 Ljubljana, Slovenia; irena.drevensek@ijs.si; 2 Faculty of Electrical Engineering, Computer Science and Information Technology, Josip Juraj Strossmayer University of Osijek, Kneza Trpimira 2B, 31000 Osijek, Croatia; 3 Faculty of Physics, University of Vienna, Boltzmanngasse 5, 1090 Vienna, Austria; mathias.fleisch@pccl.at; 4 Polymer Competence Center Leoben, AT-8700 Leoben, Austria; 5 International Sino-Slovenian Joint Research Center on Liquid Crystal Photonics, TEDA Institute of Applied Physics & School of Physics, Nankai University, Tianjin 300071, China; zxz@nankai.edu.cn; 6 J. Stefan Institute, Jamova 39, 1000 Ljubljana, Slovenia

**Keywords:** nematic liquid crystals, photoresist polymeric materials, laser-based micro structuring, diffractive optical elements

## Abstract

We present an experimental and theoretical investigation of the optical diffractive properties of electrically tuneable optical transmission gratings assembled as stacks of periodic slices from a conventional nematic liquid crystal (E7) and a standard photoresist polymer (SU-8). The external electric field causes a twist-type reorientation of the LC molecules toward a perpendicular direction with respect to initial orientation. The associated field-induced modification of the director field is determined numerically and analytically by minimization of the Landau–de Gennes free energy. The optical diffraction properties of the associated periodically modulated structure are calculated numerically on the basis of rigorous coupled-wave analysis (RCWA). A comparison of experimental and theoretical results suggests that polymer slices provoke planar surface anchoring of the LC molecules with the inhomogeneous surface anchoring energy varying in the range 5–20 μJ/m^2^. The investigated structures provide a versatile approach to fabricating LC-polymer-based electrically tuneable diffractive optical elements (DOEs).

## 1. Introduction

Diffractive optics is an important field of modern optics that deals with optical elements whose function is based on optical diffraction phenomena [1]. Various optical devices use diffractive optical elements (DOEs) to create a desired spatial profile of the optical field. Most DOEs are transparent surface relief microstructures that can affect either the amplitude or the phase of optical radiation passing through them [2,3,4,5,6]. They are standard in use as diffusers [7], beam splitters [8], beam samplers [9], beam shapers [10], axicons [11], vortex phase plates [12], etc. Lately, they have also found application in products such as 3D displays [13], consumer electronics [14], VR/AR/MR displays [15], automotive vehicles [16], and in fields such as robotics [17] or medically-required and elective surgery [18,19].

The simplest DOEs are optical diffraction gratings. These are one-dimensional periodically modulated structures used to separate different wavelengths of light in monochromators [20], spectrometers [21], and wavelength multiplexing devices [22]. They are also used to select the wavelength of output coupling in external-cavity lasers [23], modulate the transmitted pulse in optical pulse compression devices [24], and in many other optical instruments. Tunability is one of the important properties of diffraction gratings. Various tuning methods have been proposed; diffractive properties can be modulated by an applied electrical voltage [25,26,27,28], magnetic field [29,30,31], pressure, temperature, or by another electromagnetic wave [32,33,34,35,36,37]. Liquid crystals (LCs) are very suitable for the construction of tuneable optical diffraction gratings [38,39] since they possess a unique combination of mechanical softness and strong optical anisotropy that provides relatively easy control of LC molecules’ orientation. Due to the strong sensitivity of LCs to various external stimuli, it is possible to generate periodic structures built from LCs with periodically modulated stimuli, for instance, by using periodic electrode configurations [28,40] or via periodic alignment layers. The latter can be achieved either by rubbing [41,42] or by photo-alignment [43,44]. The advantage of photo-alignment over rubbing-based alignment is that its fabrication process is noncontact, which is suitable for inaccessible areas, e.g., in organic electronics [45]. Photoalignment technique is also suitable for micro-patterning of liquid crystal phases [46,47].

Liquid crystals have been used for producing various devices with appropriate desired properties, e.g., a high strength [48] or specific optical orientation [49,50]. Chiral liquid crystals exhibit a spontaneous periodic spatial modulation of optical properties [51,52]. Still, due to orientational restrictions, they are often quite difficult to incorporate into complex predefined architectures. So, to achieve more versatility, both chiral and achiral LC materials are often combined with some other optically transparent materials, for example, polymers. The polymer component, used as a scaffold to generate the desired periodic structure, is usually not sensitive to the external stimulus used to modify the LC medium. So, the variation of refractive index and consequently of optical diffractive properties is obtained by changing the orientation of the LC molecules inside the polymeric scaffold.

This work presents an experimental and theoretical investigation of the optical diffraction properties of electrically tuneable diffraction gratings fabricated from a commercial nematic LC mixture (E7) and a standard photoresist polymeric material (SU-8). The innovation of this approach is that the polymeric material is present in the volume of the grating structure and not only on its surfaces, as is the case in photoalignment-based LC gratings. The orientational structure of the LC medium, located inside the periodic polymeric scaffold as a function of an applied external electric field, is calculated by minimization of the Landau–de Gennes free energy [53,54,55]. The resulting orientational structure is then used for the calculation of optical transmission and diffraction properties. The diffraction properties are calculated by the use of rigorous coupled-wave analysis (RCWA) [56,57,58,59]. The presented methodology provides an effective tool for designing various LC-based DOEs and simulations of their operation controlled by an external electric field.

## 2. Experimental Investigation

### 2.1. Fabrication and Operation of Grating Structures

**Figure 1:**
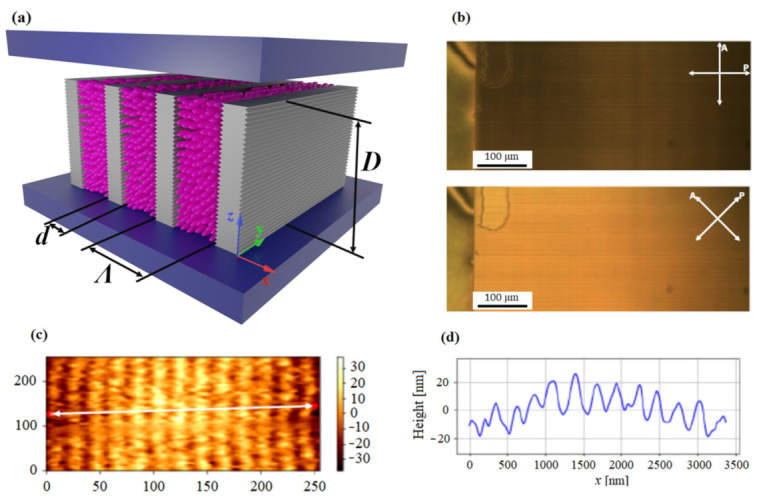
(**a**) Schematic drawing of the polymer scaffold filled with a nematic liquid crystal. The polymer scaffold is constructed as parallel polymeric ribbons fabricated from the photoresist polymer SU-8. Grey walls with the periodic surface relief structure indicate polymer ribbons, and pink ellipsoids indicate LC molecules. (**b**) Polarization optical microscopy (POM) image of the grating structure filled with the nematic LC material showing perfect LC alignment in the region of the grating structure and practically no alignment outside of the grating region. The images were captured under crossed polarizers rotated by 0°/90° (top image) and 45°/135° (bottom image) with respect to the grating lines. (**c**) Atomic force microscopy (AFM) image of the periodic surface relief structure at a sidewall of the ribbon. (**d**) The cross-sectional profile of the image along the white line indicated in (**c**).

An electrically tuneable diffraction grating is obtained by injecting a nematic LC mixture (E7, Merck Ltd., Darmstadt, Germany) into a polymer scaffold constructed from parallel polymeric ribbons. The ribbons are located on top of the conductive ITO-coated glass plate and are fabricated by direct laser writing (DLW), using a negative photoresist material (SU-8 3000, MicroChem, Westborough, MA, USA [60]) (Figure 1a). The details of the fabrication process are described elsewhere [61,62,63,64]. The ribbon assembly is covered with another ITO-coated glass plate and glued at the edges. After this, channels between the ribbons are filled with the LC material by capillary action (Figure 1b). In Figure 1a, the main parameters that determine the diffraction properties of the grating are denoted. These are the grating period *Λ*, the width of the polymer ribbons *d*, and the height of the polymer ribbons *D*. The last also corresponds to the thickness of the grating structure. Most of the experiments reported in this work were performed on gratings with *Λ* = 5 μm, *d* = 2 μm, and *D* ∼ 10 μm.

Figure 1b shows a polarization optical microscopy (POM) image of the grating structure filled with the LC medium. A very good LC alignment in the region of the grating structure and almost no alignment outside of the grating region is resolved under crossed polarizers rotated by 0°/90° (top image) and 45°/135° (bottom image) with respect to the grating lines. In-plane alignment of an LC medium between the polymer ribbons is induced by surface undulation on the sidewalls of the ribbons [63,64,65,66,67], which is schematically drawn in Figure 1a. Grey-coloured walls with the periodic surface relief structure indicate polymer ribbons.

The fs IR laser source used for the DLW method produces the periodic relief structure at the surface of the polymer ribbons due to the interference of the incident writing beam with the beam reflected from the ITO layer. This surface relief structure is visible in Figure 1c, which shows the atomic force microscopy (AFM) image of the sidewall of a single ribbon. From the cross-sectional profile taken along the white line indicated in Figure 1c, one can see that the groove depth is around 20 nm, and groove periodicity is about 250 nm (Figure 1d). The presence of the surface relief on the sidewalls of the ribbons forces the nematic director **n** to be oriented along the grooves, so as to achieve a minimum of elastic free energy of the LC medium in contact with the grooves [68]. This interfacial interaction is described by the surface anchoring energy *W*. Our previous measurements performed with a single horizontally flipped ribbon gave a value *W* = 1.3 × 10^−5^ J/m^2^ [64].

**Figure 2:**
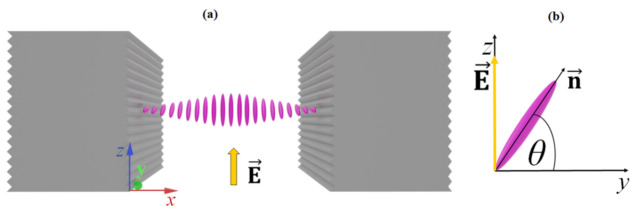
(**a**) Schematic drawing of the reorientation process of the LC molecules induced by an external electric field **E** oriented along the *z*-axis. (**b**) Local (re)orientation of the nematic director **n** under the influence of **E**, where *θ*(*x*) indicates inclination angle of **n** with respect to the *y*-axis.

When an external electric field **E** (1 kHz square-shaped, up to 10 V/μm (RMS)) is applied in the direction perpendicular to the grating structure (i.e., along the *z*-axis in Figure 1a), this causes out-of-plane LC reorientation towards the direction perpendicular to the initial orientation, as shown in Figure 2a. The reorientation process is spatially inhomogeneous, because anchoring on the surfaces of the ribbons tries to preserve the orientation of **n** parallel to the ribbons, i.e., along the *y*-axis in Figure 2a. Therefore, the electric field **E** generates a twist-type deformation between the two polymer ribbons, and the resulting orientational profile can be described as **n** = (0, cos(*θ*(*x*)), sin(*θ*(*x*))), where *θ*(*x*) is an inclination angle of **n** with respect to the *y*-axis (Figure 2b). As mentioned earlier, changing the orientation of the LC molecules causes variation of spatial modulation of the optical properties of the assembly. Hence, such a system is very suitable for the construction of electrically tuneable DOEs.

### 2.2. Optical Properties

**Figure 3:**
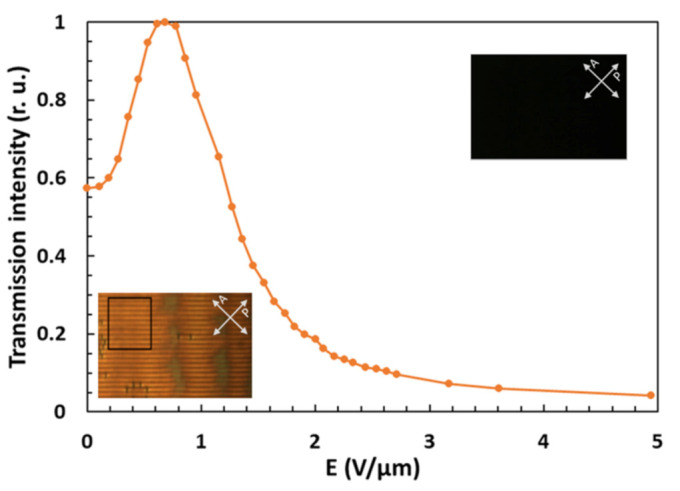
Normalized average transmission intensity as a function of the applied electric field *E*. The insets show polarization optical microscopy (POM) images of the grating structure at *E* = 0 V/μm (lower left corner) and *E* = 4.6 V/μm (upper right corner). A black rectangle denotes the region of interest (ROI) with a size of around 50 × 50 μm^2^. Arrow-ended white lines denote orientations of the polarizer (P) and the analyzer (A).

To test the tuning capability of the gratings, we first investigated their transmission properties in the POM system using objectives with a relatively large numerical aperture. The result is shown in Figure 3. The transmission axes of the crossed polarizer and analyzer were rotated by 45°/135° with respect to the grating lines. Next, the effect of the electric field on transmitted light intensity was recorded with a CCD camera (Pixelink PL-B957U, Ottawa, ON, Canada) by capturing a series of images at different strengths of the field. The selected regions of interest (ROIs) were analyzed by a program written in Python. One such ROI is indicated by a black square in the left corner inset of Figure 3. Its size is ∼50 × 50 μm^2^, which was also the typical spot size of the probe laser beam in our optical diffraction experiments.

Figure 3 shows a modification of the normalized transmission intensity as a function of the applied electric field. Before normalization, the background was subtracted, which was determined as the intensity measured when the sample was rotated such that grating lines were parallel to the direction of the polarizer. One can see that by increasing the amplitude of electric field *E*, the transmission intensity first increases and then decreases. The insets show POM images obtained for *E* = 0 V/μm (lower left corner) and *E* = 4.6 V/μm (upper right corner). Since the electric field causes out-of-plane reorientation of **n** towards the *z*-axis, and polymer ribbons tend to preserve its initial orientation along the *y*-axis, a twisted LC configuration is established.

**Figure 4:**
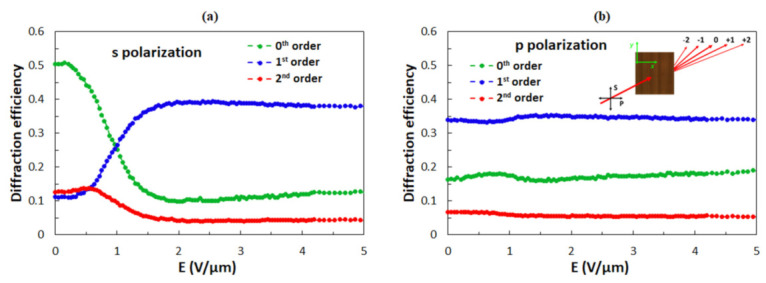
Experimentally determined diffraction efficiencies of the 0th, 1st, and 2nd diffraction orders as a function of the amplitude of the applied external electric field *E* for (**a**) s-polarized and (**b**) p-polarized incident beams. The inset in (**b**) is a schematic drawing of the diffraction experiment, in which the linearly polarized laser beam enters the sample at normal incidence with respect to the grating structure. The s- and p-polarization directions and assignment of different diffraction orders are also denoted.

Optical diffraction was probed by a linearly polarized HeNe laser with the wavelength *λ*_P_ = 633 nm propagating along the *z*-axis (Figure 1a). The polarization direction was either along the grating lines (*y*-axis, s-polarization) or perpendicular to the grating lines (*x*-axis, p-polarization). The far-field intensities of the 0th (transmission direction), 1st, and 2nd order diffraction peaks were measured by photodiodes. The intensity of the higher-order peaks was more or less negligible. Figure 4 shows the experimentally measured dependences of the diffraction efficiency of different diffraction orders on the amplitude *E* of the applied external electric field for the s-polarized (Figure 4a) and p-polarized (Figure 4b) incident beam.

The diffraction efficiency of the *i*-th diffraction order in experimental studies was calculated as:

(1) $$\eta_{i}=\frac{I_{i}}{I_{-2}{\;+\;I}_{-1}{\;+\;I}_{0}{\;+\;I}_{1}{\;+\;I}_{2}},$$

where *I*_i_ is the intensity of the selected diffraction peak, and the denominator is the sum of the intensities of the 0th, ±1st, and ±2nd diffraction order peaks.

For the s-polarized beam, in zero field, the value of *η*_0s_ is ∼0.5, the value of *η*_1s_ is ∼0.1, and the diffraction efficiency of the 2nd diffraction order *η*_2s_ is below 0.15. The application of the electric field causes a strong decrease in the diffraction efficiency of the 0th diffraction order and a profound increase of the diffraction efficiency of the 1st diffraction order. The diffraction efficiency of the 2nd diffraction order at first slightly increases and then decreases with the increasing electric field. For a p-polarized beam, in zero field, the value of *η*_0p_ is ∼0.15, the value of *η*_1p_ is ∼0.35, and the value *η*_2p_ is ∼0.05. By increasing the magnitude of **E**, the diffraction efficiency for p-polarization remains practically constant. For large electric fields, the values of diffraction efficiencies of the same diffraction orders become very similar for both polarizations.

## 3. Orientational Profile of the Liquid Crystalline Medium

### 3.1. Minimization of Free Energy

In our previous theoretical modelling of the described grating assembly, it was assumed that the LC medium between the polymer ribbons rotates homogeneously [59], i.e., independent of the *x* coordinate, which gave only a rough explanation of the observed properties. In this work, we consider an inhomogeneous reorientation of the LC molecules, i.e., we are taking into consideration a dependence of *θ*(*x*) that minimizes the free energy of the LC medium.

Due to the boundary conditions, the director **n** adjusts through the sample in order to minimize the free energy, which depends on **n** and its gradient [69], and on electric field **E**. The free energy density is given as [54]:

(2) $${f=f}^{F}-\frac{1}{2}D\cdot E,$$

where *f^F^* is the Frank elastic energy density [51], and the second term describes the coupling of **n** with external field **E**. The electric displacement vector **D** is related to the electric field **E** by $D=\varepsilon_{0}\underline{\varepsilon }\cdot E$, where $\underline{\varepsilon }$ is the dielectric tensor of LC and the external electric field is **E** = (0, 0, *E*). In accordance with Figure 2b, the free energy density is:

(3) $$f=\frac{1}{2}K_{2}{\left(\frac{\partial \theta }{\partial x}\right)}^{2}-\frac{1}{2}\varepsilon_{0}\varepsilon_{⊥}E^{2}-\frac{1}{2}\varepsilon_{0}\varepsilon_{a}E^{2}\sin^{2}\theta ,$$

where *K*_2_ is the Frank elastic constant for twist deformation [51], $\varepsilon_{0}$ is the vacuum permittivity, $\varepsilon_{⊥}$ is the dielectric constant for applied field perpendicular to **n**, and $\varepsilon_{a}$ is the dielectric anisotropy. In accordance with our experimental studies, which were performed with the conventional liquid crystal E7, we took in our calculations *K*_2_ = 7 pN [70], $\varepsilon_{⊥}={\left(n_{o}\right)}^{2}$, $\varepsilon_{a}={\left(n_{e}\right)}^{2}-{\left(n_{o}\right)}^{2}$, and the values of extraordinary and ordinary refractive indices being *n*_e_ = 1.73 and *n*_o_ = 1.52, respectively [71]. Equation (3) was used to calculate the orientational profile **n**(**r**) between two polymer (SU-8) ribbons via numerical minimization of the free energy, using Wolfram Mathematica. The space between these two ribbons was discretized into slices with width Δ*x* = *w*/(*N* − 1), where *w* is the distance between the polymer ribbons (*w* = *Λ* − *d*) and *N* is the number of discretization cells. We took the value *N* = 51, so that the thickness of the slices was around 60 nm, i.e., an order of magnitude lower than the wavelength of the probe laser beam.

To obtain the orientational profile **n**(**r**) it is necessary to minimize the free energy by considering the appropriate boundary conditions. These depend on the interactions between the LC molecules and the polymer ribbons. This is described by the Rapini–Papoular surface energy density [72]

(4) $$f^{s}=-\frac{1}{2}W{\left(n_{s}\cdot n\right)}^{2},$$

where *W* is the anchoring strength of the surface and **n_s_** ($n_{s}={\hat{e}}_{y}$) is the preferred direction of **n** at the surface.

To solve the problem analytically, we considered two limiting cases—a high and a low electric field, as in our previous work [55]. In the limit of the high electric field, it was assumed that *θ*(*x*) is close to *π*/2, while in the limit of the low electric field, it was assumed that *θ*(*x*) is close to zero. By assuming a twist-type deformation in the limit of the high electric field, Equation (3) becomes:

(5) $$f\;\approx \frac{1}{2}K_{2}{\left(\frac{\partial \theta }{\partial x}\right)}^{2}+\frac{1}{2}\varepsilon_{0}\varepsilon_{a}E^{2}{\left(\theta -\frac{\pi }{2}\right)}^{2},$$

where constant terms and terms of the Taylor series higher than *O*(*θ*^2^) are omitted.

In order to find the equilibrium solution for *θ*(*x*), it is necessary to minimize the free energy using the Euler–Lagrange formalism [54]. The general solution is:

(6) $$\theta (x)=\frac{\pi }{2}+C_{1}e^{qx}+C_{2}e^{-qx},$$

where

(7) $$q^{2}{=E}^{2}\frac{\varepsilon_{0}\varepsilon_{a}}{K_{2}}.$$

By applying the boundary condition, which is related to the requirement that the equilibrium solution for director **n** at the surface must satisfy the anchoring condition, the values of constants *C*_1_ and *C*_2_ are obtained. The boundary condition is [54]:

(8) $$K_{2}\frac{\partial \theta }{\partial x}\nu +\frac{\partial f^{s}}{\partial \theta }=0,$$

where *ν* is −1 at *x* = 0 and +1 at *x* = *w*. For strong anchoring (*W* >> *K*_2_/*w*), the equilibrium solution is:

(9) $$\theta (x)=\frac{\pi }{2}-\frac{\frac{\pi }{2}}{1+\xi q\tanh(qw/2)}\frac{\cosh(q(x-w/2))}{\cosh(qw/2)},$$

while for weak anchoring (*W* << *K*_2_/*w*), it is:

(10) $$\theta (x)=\frac{\pi }{2}-\sqrt{\frac{3}{2}\left(1-\xi q\tanh(qw/2)\right)}\frac{\cosh(q(x-w/2))}{\cosh(qw/2)},$$

where *ξ = K*_2_*/W*. Equation (10) is valid as long as ξqtanh(qw/2)≤1. If ξqtanh(qw/2) is above 1, then *θ*(*x*) is *π*/2 everywhere, i.e., the relation (10) is valid for external electric fields that satisfy the condition using the Euler–Lagrange formalism [54]. The general solution is:

(11) $$\frac{E}{W}\sqrt{K_{2}\varepsilon_{0}\varepsilon_{a}}\tanh\left(\frac{w}{2}E\sqrt{\frac{\varepsilon_{0}\varepsilon_{a}}{K_{2}}}\right)\leq 1,$$

otherwise, *θ*(*x*) is *π*/2 everywhere.

The limits of strong and weak anchoring are taken into account, because high electric fields and strong anchoring strengths have opposite effects. Strong electric fields tend to rotate the director **n** in the direction of the electric field **E**, while strong anchoring strengths tend to preserve the director **n** in the initial orientation dictated by the surface of polymer slices.

For the limit of low electric fields and the assumption of twist type deformation, the free energy (3) is:

(12) $$f\approx \frac{1}{2}K_{2}{\left(\frac{\partial \theta }{\partial x}\right)}^{2}-\frac{1}{2}\varepsilon_{0}\varepsilon_{a}E^{2}\theta^{2},$$

where constant terms and terms of the Taylor series higher than *O*(*θ*^2^) are omitted. The solution for *θ*(*x*) is described in [73] and it is:

(13) $$\theta (x)=\delta \cdot A\cos(E_{F}\sqrt{\frac{\varepsilon_{0}\varepsilon_{a}}{K_{2}}}(x-w/2)),$$

where *δ* and *A* are

(14) $$\delta =\sqrt{\frac{E}{E_{F}}-1},$$

(15) $$A=2\sqrt{\frac{\left(1-\eta \right)\left(V_{F}^{2}+\beta^{2}\right)\left(V_{F}^{2}+2\beta +\beta^{2}\right)}{\left(\beta^{4}+2\beta^{3}+2\beta^{2}V_{F}^{2}-2{\beta V}_{F}^{2}+V_{F}^{4}\right)+4\beta \eta \left(V_{F}^{2}-\beta^{2}-2\beta \right)}},$$

where *β* = *w·W/K*_2_, $\eta =1-\varepsilon_{⊥}/\varepsilon_{∥}$, $V_{F}=\pi w/(w+2\xi )$, *ξ* = *K*_2_*/W* as before, and *E*_F_ is the threshold field of the Fréedericksz transition. Its value is given by

(16) $$E_{F}=\frac{\pi }{w+2\xi }\sqrt{\frac{K_{2}}{\varepsilon_{0}\varepsilon_{a}}}.$$

Equation (13) is valid for the electric field amplitudes *E* that are slightly above *E*_F_. If *E* is below *E*_F_, then *θ*(*x*) is zero. In the limit of low electric fields, the limit of strong anchoring is not relevant since low electric fields and strong anchoring strengths have similar effects. For both of them, the director **n** only slightly deviates from its initial orientation.

### 3.2. Comparison of Analytical and Numerical Results

**Figure 5:**
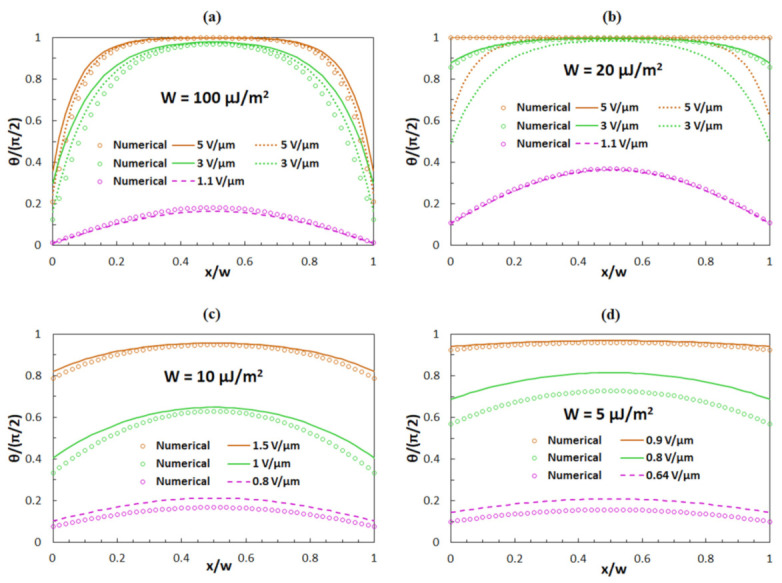
Numerical and analytical results for *θ*(*x*) for anchoring strengths of (**a**) *W* = 100 μJ/m^2^, (**b**) *W* = 20 μJ/m^2^, (**c**) *W* = 10 μJ/m^2^ and (**d**) *W* = 5 μJ/m^2^. Circles correspond to the numerical solution, dotted lines to Equation (9), solid lines to Equation (10) and dashed lines to Equation (13). The results are given for three different values of the electric field.

Based on the above presented theoretical considerations, a comparison of numerical and analytical results for *θ*(*x*) can be made. In Figure 5, circles correspond to the numerical results, while dotted lines correspond to the analytical results associated with Equation (9), solid lines to the analytical results obtained by Equation (10), and dashed lines to the analytical results obtained by Equation (13). The results for *θ*(*x*) are given for anchoring strengths *W* of 100 (Figure 5a), 20 (Figure 5b), 10 (Figure 5c), and 5 μJ/m^2^ (Figure 5d).

In Figure 5a,b, one can notice that for the anchoring strength *W* = 100 μJ/m^2^ and electric fields *E* = 5 V/μm and *E* = 3 V/μm, the analytical results obtained by Equation (9) exhibit a much better agreement with the numerical results than the analytical results obtained by Equation (10). For the anchoring strength *W* = 20 μJ/m^2^, the situation is just the opposite. So, for the investigated system, the anchoring strength *W* = 100 μJ/m^2^ can be considered a strong anchoring, while the anchoring strength *W* = 20 μJ/m^2^ can be considered a weak anchoring, and both fields can be considered high electric fields. Yet, according to the ratio *K*_2_/*w*, which defines the regimes of strong and weak anchoring (*K*_2_/*w* = 2.3 μJ/m^2^), the anchoring energy *W* = 20 μJ/m^2^ should not yet be a weak anchoring. However, we note that the dielectric anisotropy should also be taken into consideration. For the electric field *E* = 1.1 V/μm (see Figure 5a,b), the analytical results obtained by Equation (13) give relatively good matching. This field is slightly above the threshold field *E*_F_ ∼1 V/μm for the anchoring strength *W* = 100 μJ/m^2^ and also above *E*_F_ ∼0.91 V/μm for the anchoring strength *W* = 20 μJ/m^2^. So, for the investigated system, these fields can be considered low electric fields.

With further decrease of *W*, the difference between *E*_F_ and *E,* above which *θ*(*x*) is everywhere equal to *π*/2, becomes lower and lower. The value of critical field *E,* above which *θ*(*x*) = *π*/2, for anchoring strengths *W* = 10 μJ/m^2^ (Figure 5c) is around 1.6 V/μm, and for anchoring strengths *W* = 5 μJ/m^2^ (Figure 5d) it is around 0.95 V/μm. For values of *E* (Figure 5c,d; brown colour) that are slightly below those critical fields, analytical results obtained by Equation (10) exhibit good agreement with the numerical results, while for values of *E* (Figure 5c,d; purple colour) that are slightly above *E*_F_, the analytical results obtained by Equation (13) exhibit a slight deviation from the numerical results. For example, for very low anchoring strengths *W* = 1 µJ/m^2^, the difference between the threshold field *E*_F_ and the field *E,* above which *θ*(*x*) is everywhere *π*/2, is very small. The threshold field is *E*_F_ ≈ 0.32 V/μm and the reorientation angle *θ*(*x*) is *π*/2 for electric fields *E* > 0.35 V/μm for numerical and analytical determination of the reorientation angle *θ*(*x*). Therefore, it is much more challenging to determine which approximation is valid for which electric fields.

## 4. Theoretical Analysis of Optical Properties

### 4.1. Calculation of Optical Transmission Properties

In the theoretical analysis of optical transmission properties, the LC (E7) slices are described as an optically uniaxial material with principal refractive indices *n*_e_ = 1.73 and *n*_o_ = 1.52. Each slice is divided into *N =* 51 sub-slices, characterized by the orientational profile *θ*(*x*), which depends on the external electric field *E*, as described earlier. The transmission intensity of each sub-slice, for crossed polarizers oriented at 45°/135° with respect to the *y*-axis, was calculated by using the relation [74]:

(17) $$T=\sin^{2}\left((n_{eff}-n_{o})\frac{\pi D}{\lambda }\right),$$

where *n_eff_* is given by:

(18) $$n_{eff}=\frac{1}{\sqrt{{\left(\frac{\cos\theta }{n_{e}}\right)}^{2}+{\left(\frac{\sin\theta }{n_{o}}\right)}^{2}}}$$

and *λ* is the wavelength of the illumination light, while *D* is the thickness of the grating structure. The average transmission intensity over several grating periods, which is the quantity that was measured in the experiments (Figure 3), is obtained by summing the transmission intensity of all LC sub-slices and dividing this sum by the number of slices *N*.

**Figure 6:**
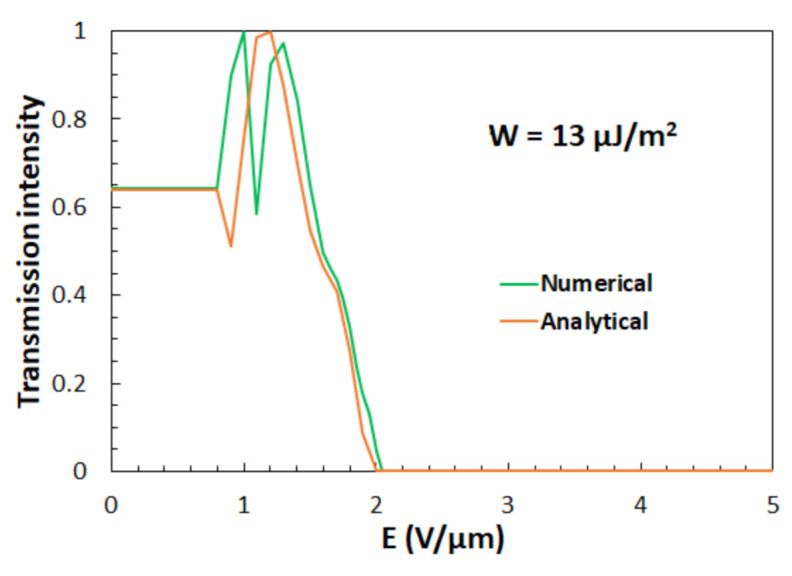
Calculated normalized transmission intensity for *λ* = 550 nm as a function of an applied electric field *E* for the anchoring strength *W* = 13 μJ/m^2^ and grating thickness *D* = 8.45 μm. The green line refers to the calculation in which *θ*(*x*) was determined numerically and the orange line to the calculation in which *θ*(*x*) was determined analytically. For the latter, Equation (10) was used for *E* > 1 V/μm, while Equation (13) was used for *E* ≤ 1 V/μm.

In the calculation of the transmission intensity, we used the thickness of the grating *D* = 8.45 μm (with the grating period *Λ* = 5 μm and the width of the LC slices *w* = 3 μm) [59] and the wavelength of the illumination light *λ* = 550 nm, since white light was used in the experiment. Figure 6 shows the average transmission intensity as a function of the applied electric field *E* obtained for the anchoring strength *W* = 13 μJ/m^2^ [64]. A comparison of the results for which the values of *θ*(*x*) were determined numerically (green line) and analytically by Equation (10) (orange line) are given for *E* > 1 V/μm, while for *E* ≤ 1 V/μm Equation (13) was used.

Similar to our experimental results (see Figure 3), one can observe that, by increasing electric field *E,* transmission intensity first increases, then reaches a maximum, and afterward, with further increase of the electric field, it decreases to zero.

Deviations between the theoretical and the experimental results (Figure 3) are attributed to a spatial variation of surface anchoring strength *W* within the selected ROI. Stronger anchoring would cause weaker reorientation of the director **n** from its initial direction along the *y*-axis for all the applied electric fields so that the transmission intensity would fall to zero slower. Besides this, some imperfections in the alignment process, which are often encountered in these experiments, smear out the threshold behavior. In addition, some other grating parameters may differ from region to region, too. Nevertheless, the above-described comparison confirms the primary idea that the field-induced reorientation of the director field **n**(**r**) in the investigated assembly is a twist-type reorientation.

### 4.2. Calculation of Optical Diffraction Properties

A schematic drawing of the diffraction system analyzed by numerical calculations is depicted in the inset of Figure 4b. The SU-8 polymer slices, described as optically isotropic material with refractive index *n*_p_ = 1.57 [60], are combined with the LC slices, described as optically uniaxial material. The incident optical beam is propagating along the *z*-axis and is linearly polarized either along the *y*-axis (s-polarization) or along the *x*-axis (p-polarization), as mentioned in Section 2.2.

**Figure 7:**
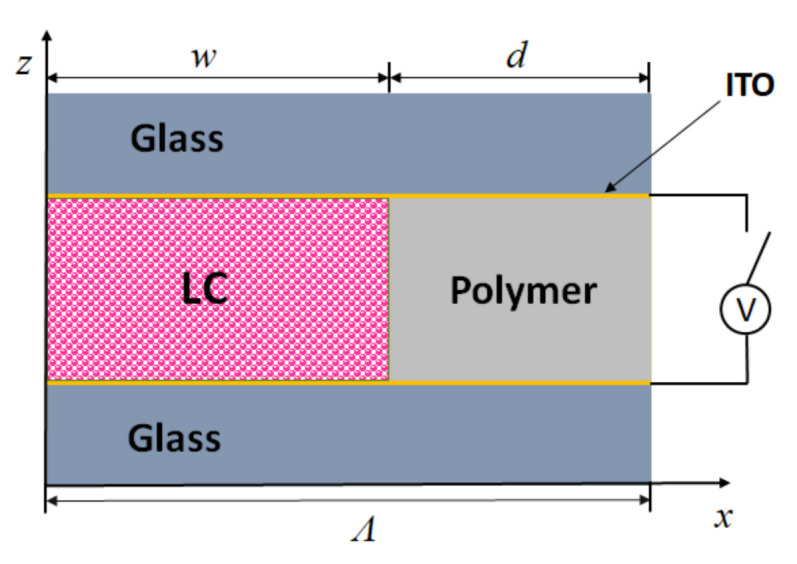
Schematic drawing of the cross-section of a unit cell used for RCWA calculations with the S^4^ solver.

According to Gaylord et al. [75], the grating structures that are the subject of our research belong neither to the category of thin (Raman–Nath) nor to the category of thick (Bragg) gratings, but can be classified, somewhere, between those two regimes. Therefore, in order to obtain a suitable description of their diffraction characteristics, a numerical method based on coupled-wave theory was developed [56]. The rigorous coupled-wave analysis (RCWA) is a semi-analytical method, which is used to describe light propagation in optical grating structures with various grating periodicities and thicknesses. The basic idea of this method is described in our previous work [55]. To perform the calculation described in this work, we used the Stanford Stratified Structure Solver (S^4^) [76], which is the freely available electromagnetic solver for solving Maxwell’s equations in layered periodic structures, based on the RCWA. A schematic drawing of the cross-section of a unit cell used for such numerical calculations is shown in Figure 7.

The width of the unit cell in the *x*-direction was *Λ* = 5 μm. The individual regions were defined by their thickness and respective relative permittivity $\varepsilon_{r}$ for isotropic materials and by dielectric tensor $\underline{\varepsilon }$ for the anisotropic LC medium. For relative permittivity of the polymer medium, we took $\varepsilon_{p}={\left(1.57\right)}^{2}$ [60]. Since the external electric field **E**, oriented parallel to the *z*-axis, causes reorientation of the optical axis of the LC medium in the *yz* plane, the LC medium was described by a dielectric tensor:

(19) $$\underline{\varepsilon }=\left(\begin{array}{ccc} \varepsilon_{⊥} & 0 & 0\\ 0 & \varepsilon_{⊥}{+\varepsilon }_{a}\cos^{2}\theta (x) & \varepsilon_{a}\sin\theta (x)\cos\theta (x)\\ 0 & \varepsilon_{a}\sin\theta (x)\cos\theta (x) & \varepsilon_{⊥}{+\varepsilon }_{a}\sin^{2}\theta (x) \end{array}\right),$$

where *θ*(*x*) is the angle of reorientation with respect to the *y*-axis (see Figure 2b), which depends on the field magnitude *E*. In the numerical calculation of diffraction efficiencies as a function of *E*, we used the analytical and numerical results for *θ*(*x*) presented in Section 3.2.

**Figure 8:**
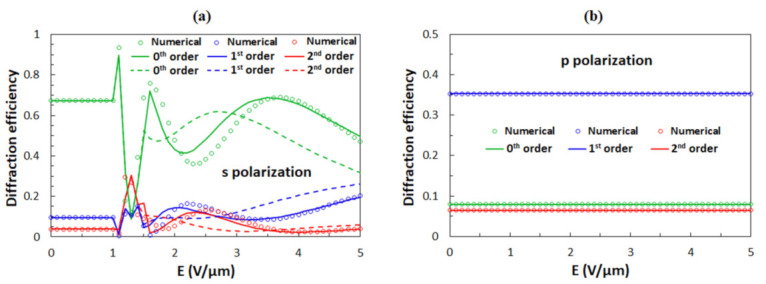
Diffraction efficiencies of the 0th, 1st, and 2nd diffraction orders as a function of the applied external electric field *E* for (**a**) the s-polarized and (**b**) the p-polarized incident beams. The anchoring strength is *W* = 100 μJ/m^2^. Circles are obtained with the numerical solution for *θ*(*x*), while solid lines are results for *θ*(*x*) obtained by Equation (9) and dashed lines for *θ*(*x*) obtained by Equation (10). In both analytical cases, for *E* ≤ 1.4 V/μm, results for *θ*(*x*) are obtained by Equation (13).

Figure 8 gives the numerical calculations of the diffraction efficiency *η* of the 0th, 1st, and 2nd diffraction orders as a function of *E* for the anchoring strength *W* = 100 μJ/m^2^. The curves for which *θ*(*x*) was obtained by Equation (9) are given as solid lines and those for which *θ(x)* was obtained by Equation (10) are given as dashed lines. In both cases, for *E* ≤ 1.4 V/μm, Equation (13) was used. One can notice that the analytical results for strong anchoring obtained by Equation (9) give better agreement with the numerical results than the analytical results for weak anchoring obtained by Equation (10). This is in line with the explanation in Section 3.2.

The comparison of numerical calculations with experimental data for s-polarized beam (Figure 4a) reveals that the agreement is not very good. Numerical calculations predict a profoundly oscillatory behavior for *η*_0s_ and *η*_1s_, while in the experiments, *η*_0s_ at first decreases to a minimum value of around 0.1 and then only slightly increases, while *η*_1s,_ at first, increases to around 0.4 and then only slightly decreases. One possible reason for this discrepancy is the interaction of the LC medium with the top and the bottom surfaces (anchoring at ITO-coated glass plates), which is fully neglected in our analysis. Another, and in our opinion a more probable reason, could be spatial inhomogeneity of the reorientation process due to special variation of the anchoring strength *W*. The latter is examined in the current paper.

The diffraction efficiency for a p-polarized beam is not sensitive to *E* (see Figure 8b). This is because the *ε*_11_ component of the dielectric tensor, which is relevant for this polarization, does not change during the out-of-plane reorientation process. By comparing the calculation results with the experimental data for p-polarization shown in Figure 4b, one can notice that the agreement between the calculation and the experiment is fairly good. Experimentally obtained values of *η*_1p_ and *η*_2p_ are slightly different than in the calculation, but this discrepancy could be reduced by changing the grating parameters. However, we decided to use the same grating parameters as in our previous work [59].

**Figure 9:**
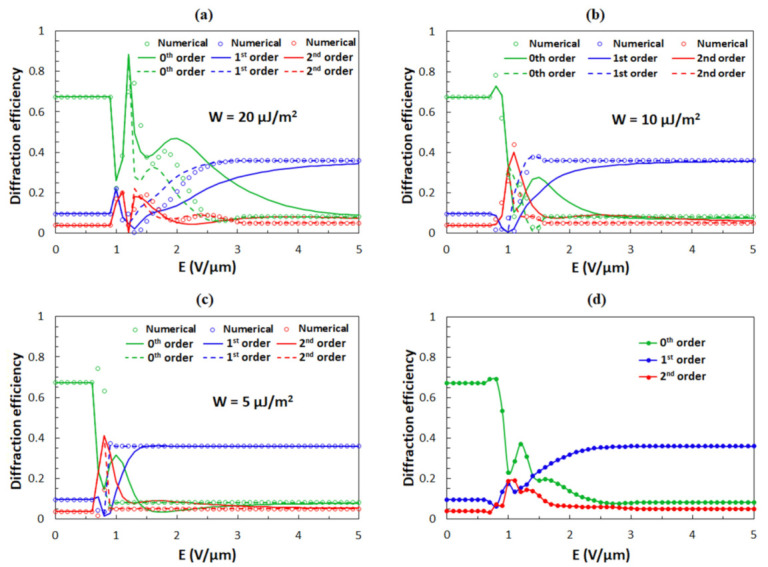
Diffraction efficiencies of the 0th, 1st, and 2nd diffraction orders as a function of the applied external electric field *E* for the s-polarized incident beam. The anchoring strength is (**a**) *W* = 20 μJ/m^2^, (**b**) *W* = 10 μJ/m^2^, and (**c**) *W* = 5 μJ/m^2^. Circles are obtained with the numerical solution for *θ*(*x*), while solid lines are results for *θ*(*x*) obtained by Equation (9) and dashed lines for *θ*(*x*) obtained by Equation (10). In analytical results *θ*(*x*) were obtained by Equation (13) for anchoring strength *W* = 20 μJ/m^2^ up to 1.2 V/μm, for anchoring strength *W* = 10 μJ/m^2^ up to 1 V/μm, and for anchoring strength *W* = 5 μJ/m^2^ up to 0.7 V/μm. (**d**) The average diffraction efficiencies of the 0th, 1st, and 2nd diffraction orders as a function of the applied external electric field *E* for anchoring strengths *W* = 20, 15, 10, 5 μJ/m^2^, for which the reorientation angles were determined numerically.

With decreasing anchoring strength *W*, the oscillations in the dependence of *η*_s_ as a function of *E* become less profound. Figure 9 shows the numerical calculations of the diffraction efficiencies of the 0th, 1st, and 2nd diffraction orders for *W* = 20, 10, and 5 μJ/m^2^. One can notice that curves for which *θ*(*x*) was obtained by Equation (10) give better agreement with the numerical results than the curves for which *θ*(*x*) was obtained by Equation (9). Diffraction efficiencies for s- (Figure 9) and p-polarization (Figure 8b) become similar at high field strengths. From the comparison of the numerical calculations (Figure 9) and the experimental data (Figure 4), we conclude that the average anchoring strength *W* between the surface of the polymer ribbons and the LC molecules in the experimentally investigated structures is slightly above 10 μJ/m^2^.

Since the grooves on the sidewalls of the polymer ribbons have differing depths (see Figure 1d), it is expected that the anchoring energy is inhomogeneous, i.e., the value of the anchoring strength *W* is not equal over the entire surface area of the polymer ribbons. To take this into account, we calculated diffraction efficiencies of the 0th, 1st, and 2nd diffraction orders as a function of *E* averaged over four different anchoring strengths *W* = 20, 15, 10, and 5 μJ/m^2^ (Figure 9d), which are the values around the previously measured value *W* = 13 μJ/m^2^ [64]. Figure 9d shows the corresponding result for the case in which the reorientation angle *θ*(*x*) was determined numerically. The averaging causes a smearing of oscillatory features and consequently gives the dependence that much better matches the experimental data. From this result, it can be concluded that in our grating assembly, the anchoring strength is varying on the microscale such that its value is changing between 5 and 20 μJ/m^2^.

Due to the relatively low anchoring strength of LC media on the ITO surface [77], in the present simulations we neglected entirely the LC anchoring at the top and the bottom ITO-coated glass surfaces. Further improvements can hence be obtained by also considering interactions at those interfaces. For this purpose, it would be necessary to extend the existing analysis by considering the reorientation process described as *θ* = *f* (*x*, *z*), which would require modelling, with a multilayer RCWA system, that is computationally more demanding. There also exist some general disadvantages of the RCWA that pose some limitations on the modelling of the presented structures. For instance, RCWA is not efficient for aperiodic structures, it is not spatially discretized, and for p-polarization it is restricted to lamellar profiles [78,79]. Modelling of the grating structures with tilted polymer ribbons would also require “cutting” of the grating into several layers, etc.

## 5. Discussion and Conclusions

The experimental and theoretical analysis of the transmission and diffraction properties of electrically tuneable LC-polymer diffraction gratings were presented. The investigated periodically modulated structure was based on periodic slices of the nematic liquid crystal E7 and the photoresist polymer SU-8. These two materials were selected because their optical and electric properties are well known. However, the flexibility of the investigated grating assemblies lies in the fact that there are practically no restrictions on the selection of the polymer and the LC medium. Therefore, one can select the substances that are optimal for the required operating conditions.

Since the theoretical description of electrically tuneable gratings presented in this work and the theoretical description of magnetically tuneable gratings presented in our previous work [55] both agreed very well with our experimental results, we believe that the developed methodology can also be used to analyze the combined tuning of such grating structures with both electric and magnetic fields. For this purpose, a standard nematic LC material would have to be replaced with a ferromagnetic nematic LC mixture [80]. One field could then be used to cause reorientation, for instance, into the twist-type configuration as investigated in this paper, and another field could be used to return the LC back to the initial homogeneous configuration. Such two-field driven active tuning is expected to provide much shorter switching times than in standard LC-based gratings, which combine only one active switching process with a passive relaxation guided by the boundary conditions. Our work is expected to stimulate experimental as well as theoretical research in this area.

Other interesting challenges are 2D and 3D LC-polymer-based diffractive structures. Based on preliminary experiments with 2D LC-polymer gratings [81,82,83,84], which allow multistable LC orientational profiles within a unit cell, it is necessary to construct an RCWA-based model to simulate the diffraction properties of such structures. This will enable precise identification and mapping of (meta)stable LC configurations within polymeric square wells. Also, in this case, an electric or a magnetic field or a combination of both can be used to switch between different (meta)stable configurations, which opens up possibilities for their applications in devices such as powerless LCD units.

## Data Availability

The data presented in this study are available on link: https://drive.google.com/drive/folders/1fM6zo8CfD8qGZRykaTly_rpk7sRwEHoQ?usp=sharing (accessed on 1 June 2021). Additional data are available on request from the corresponding author.

## References

[1] O’Shea D.C., Suleski T.J., Kathman A.D., Prather D.W. (2003). Diffractive Optics: Design, Fabrication, and Test.

[2] Soskind Y.G. (2011). Field Guide to Diffractive Optics. Field Guide to Diffractive Optics.

[3] Huang K., Shi P., Kang X.-L., Zhang X., Li Y.-P. (2010). Design of DOE for generating a needle of a strong longitudinally polarized field. Opt. Lett..

[4] Cojoc D., Di Fabrizio E., Businaro L., Cabrini S., Romanato F., Vaccari L., Altissimo M. (2002). Design and fabrication of diffractive optical elements for optical tweezer arrays by means of e-beam lithography. Microelectron. Eng..

[5] Komanduri R.K., Oh C., Escuti M.J. (2009). 34.4L: Late-News Paper: Polarization Independent Projection Systems Using Thin Film Polymer Polarization Gratings and Standard Liquid Crystal Microdisplays. SID Symp. Dig. Tech. Pap..

[6] Seo E., Kee H.C., Kim Y., Jeong S., Choi H., Lee S., Kim J., Komanduri R.K., Escuti M.J. (2011). 39.2: Polarization Conversion System Using a Polymer Polarization Grating. SID Symp. Dig. Tech. Pap..

[7] Parikka M., Kaikuranta T., Laakkonen P., Lautanen J., Tervo J., Honkanen M., Kuittinen M., Turunen J. (2001). Deterministic diffractive diffusers for displays. Appl. Opt..

[8] De Sterke C.M., van der Laan C.J., Frankena H.J. (1983). Non-polarizing beam splitter design. J. Appl. Opt..

[9] Cao B.X., Hoang P.L., Ahn S., Kim J., Sohn H., Noh J. (2016). Real-time detection of focal position of work piece surface during laser processing using diffractive beam samplers. Opt. Lasers Eng..

[10] Katz S., Kaplan N., Grossinger I. (2018). Using Diffractive Optical Elements: DOEs for beam shaping-fundamentals and applications. Laser Tech. J..

[11] Kizuka Y., Yamauchi M., Matsuoka Y. (2008). Characteristics of a laser beam spot focused by a binary diffractive axicon. Opt. Eng..

[12] Yuan X.-C., Lin J., Bu J., Burge R.E. (2008). Achromatic design for the generation of optical vortices based on radial spiral phase plates. Opt. Express.

[13] Wang Q.-H., Ji C.-C., Li L., Deng H. (2016). Dual-view integral imaging 3D display by using orthogonal polarizer array and po-larization switcher. Opt. Express.

[14] Mi L., Chen C.P., Lu Y., Zhang W., Chen J., Maitlo N. (2018). Design of lensless retinal scanning display with diffractive optical element. Opt. Express.

[15] Kress B.C. (2020). Optical Architectures for Augmented-, Virtual-, and Mixed-Reality Headsets.

[16] Khan M.S., Saeed W.M., Roth B., Lachmayer R. (2020). Diffractive optics based automotive lighting system. A rear end lamp design for communication between road users. Adv. Opt. Technol..

[17] Dahlke D., Geßner M., Meißner H., Stebner K., Grießbach D., Berger R., Börner A. (2019). Calibrating photogrammetric airborne camera system with diffractive optical elements. Int. Arch. Photogramm. Remote Sens. Spat. Inf. Sci..

[18] Kern C., Speck U., Riesenberg R., Reble C., Khazaka G., Zieger M., Kaatz M., De Gregorio M., Fischer F. (2021). Mobile snapshot hyperspectral imaging device for skin evaluation using diffractive optical elements. Ski. Res. Technol..

[19] Fei B. (2020). Hyperspectral imaging in medical applications. Data Handling Sci. Technol..

[20] Hettrick M.C. (1992). Surface normal rotation: A new technique for grazing-incidence monochromators. Appl. Opt..

[21] Kneubühl F. (1969). Diffraction Grating Spectroscopy. Appl. Opt..

[22] Laude J.-P., Lerner J.M. Wavelength Division Multiplexing/Demultiplexing (WDM) Using Diffraction Gratings. Proceedings of the 28th Annual Technical Symposium.

[23] Harvey K.C., Myatt C.J. (1991). External-cavity diode laser using a grazing-incidence diffraction grating. Opt. Lett..

[24] Treacy E. (1969). Optical pulse compression with diffraction gratings. IEEE J. Quantum Electron..

[25] James S., Tatam R. (2003). Optical fibre long-period grating sensors: Characteristics and application. Meas. Sci. Technol..

[26] Fattal D., Peng Z., Tran T., Vo S., Fiorentino M., Brug J., Beausoleil R.G. (2013). A multi-directional backlight for a wide-angle, glasses-free three-dimensional display. Nat. Cell Biol..

[27] Tien C.-L., Lin R.-J., Kang C.-C., Huang B.-Y., Kuo C.-T., Huang S.-Y. (2019). Electrically Controlled Diffraction Grating in Azo Dye-Doped Liquid Crystals. Polymers.

[28] Huang S.-Y., Huang B.-Y., Kang C.-C., Kuo C.-T. (2020). Diffraction and Polarization Properties of Electrically–Tunable Nematic Liquid Crystal Grating. Polymers.

[29] Chen C.Y., Hsieh C.F., Lin Y.F., Pan R.P., Pan C.L. (2004). Magnetically tunable room-temperature 2π liquid crystal terahertz phase shifter. Opt. Express.

[30] Zhang L., Fan Y., Liu H., Han X., Lu W., Tao Z.Y. (2018). A magnetically tunable non-Bragg defect mode in a corrugated wave-guide filled with liquid crystals. Phys. Lett. A.

[31] Cao Y., Wang P.-X., D’Acierno F., Hamad W.Y., Michal C.A., MacLachlan M.J. (2020). Tunable Diffraction Gratings from Bio-sourced Lyotropic Liquid Crystals. Adv. Mater..

[32] Ono H., Takahashi F., Emoto A., Kawatsuki N. (2005). Polarization holograms in azo dye-doped polymer dissolved liquid crystal composites. J. Appl. Phys..

[33] Warner M., Terentjev E.M. (2007). Liquid Crystal Elastomers.

[34] Zhao Y., Zhao Y., Ikeda T. (2009). Tunable diffraction gratings based on azobenzene polymers and liquid crystals. Smart Light Responsive Materi-als—Azobenzene-Containing Polymers and Liquid Crystals.

[35] Gregorc M., Li H., Domenici V., Drevenšek-Olenik I. (2012). Tunable photonic structures from liquid crystal elastomers. Proc. SPIE.

[36] Bošnjaković D., Gregorc M., Li H., Čopič M., Domenici V., Drevenšek-Olenik I. (2018). Mechanical Manipulation of Diffractive Properties of Optical Holographic Gratings from Liquid Crystalline Elastomers. Appl. Sci..

[37] Nieborek M., Rutkowska K., Woliński T.R., Bartosewicz B., Jankiewicz B., Szmigiel D., Kozanecka-Szmigiel A. (2020). Tunable Polarization Gratings Based on Nematic Liquid Crystal Mixtures Photoaligned with AzoPolymer-Coated Substrates. Crystals.

[38] Zola R.S., Bisoyi H.K., Wang H., Urbas A.M., Bunning T.J., Li Q. (2019). Dynamic Control of Light Direction Enabled by Stimu-li-Responsive Liquid Crystal Gratings. Adv. Mater..

[39] Chen R., Lee Y., Zhan T., Yin K., An Z., Wu S. (2019). Multistimuli-responsive self-organized liquid crystal Bragg gratings. Adv. Opt. Mater..

[40] Algorri J.F., Morawiak P., Zografopoulos D.C., Bennis N., Spadlo A., Rodríguez-Cobo L., Jaroszewicz L.R., Sánchez-Pena J.M., López-Higuera J.M. (2020). Multifunctional light beam control device by stimuli-responsive liquid crystal micro-grating struc-tures. Sci. Rep..

[41] Muravsky A., Agabekov V., Zhavnerko G., Mahilny U., Stankevich A. (2010). P-123: Patterned Rubbing Alignment Technology. SID Symp. Dig. Tech. Pap..

[42] Chen J., Bos P.J., Vithana H., Johnson D.L. (1995). An electro-optically controlled liquid crystal diffraction grating. Appl. Phys. Lett..

[43] Rutkowska K., Chychłowski M., Kwasny M., Ostromęcka I., Piłka J., Laudyn U.A. (2017). Light propagation in periodic photonic structures formed by photo-orientation and photo-polymerization of nematic liquid crystals. Opto-Electron. Rev..

[44] Shen Y., Xu Y.-C., Ge Y.-H., Jiang R.-G., Wang X.-Z., Li S.-S., Chen L.-J. (2018). Photoalignment of dye-doped cholesteric liquid crystals for electrically tunable patterns with fingerprint textures. Opt. Express.

[45] Chigrinov V., Kozenkov V.M., Kwok H.-S. (2008). Photoalignment of Liquid Crystalline Materials.

[46] Pan S., Ho J.Y.-L., Chigrinov V.G., Kwok H.S. (2018). Novel Photoalignment Method Based on Low-Molecular-Weight Azobenzene Dyes and Its Application for High-Dichroic-Ratio Polarizers. ACS Appl. Mater. Interfaces.

[47] Ji W., Wei B.-Y., Chen P., Hu W., Lu Y.-Q. (2017). Optical field control via liquid crystal photoalignment. Mol. Cryst. Liq. Cryst..

[48] Harper C.A. (2000). Modern Plastics Handbook.

[49] Castellano J.A. (2005). Liquid Gold—The Story of Liquid Crystal Displays and the Creation of an Industry.

[50] Yang D.-K., Wu S.-T. (2014). Fundamentals of Liquid Crystal Devices.

[51] De Gennes P.G., Prost J. (1993). The Physics of Liquid Crystals.

[52] Vicari L. (2003). Optical Applications of Liquid Crystals.

[53] Sebastián N., Osterman N., Lisjak D., Čopič M., Mertelj A. (2018). Director reorientation dynamics of ferromagnetic nematic liquid crystals. Soft Matter.

[54] Stewart I.W. (2004). The Static and Dynamic Continuum Theory of Liquid Crystals: A Mathematical Introduction.

[55] Bošnjaković D., Sebastián N., Drevenšek-Olenik I. (2020). Magnetically Tunable Liquid Crystal-Based Optical Diffraction Gratings. Polymers.

[56] Moharam M.G., Gaylord T.K. (1981). Rigorous coupled-wave analysis of planar-grating diffraction. J. Opt. Soc. Am..

[57] Kogelnik H. (1969). Coupled Wave Theory for Thick Hologram Gratings. Bell Syst. Tech. J..

[58] Moharam M.G., Gaylord T.K., Grann E.B., Pommet D.A. (1995). Formulation for stable and efficient implementation of the rigorous coupled-wave analysis of binary gratings. J. Opt. Soc. Am. A.

[59] Fleisch M., Gao S., Bošnjaković D., Zhang X., Rupp R.A., Drevenšek-Olenik I. (2019). Laser-written polymeric scaffolds for micro-patterned liquid crystal alignment. Liq. Cryst..

[60] MicroChem SU-8 3000 Data Sheet. http://microchem.com/pdf/SU8%203000%20Data%20Sheet.pdf.

[61] Zhang X., Xu J., Li W., Drevensek-olenik I., Cui W., Shi B., Wang Z., Wu Q., Kong Y. (2018). Micro/Nano Region Liquid Crystal Alignment Method and System Thereof Based on Laser Direct Writing. Nankai University. CN Patent.

[62] Zhou X., Hou Y., Lin J. (2015). A review on the processing accuracy of two-photon polymerization. AIP Adv..

[63] Lee C.H., Yoshida H., Miura Y., Fujii A., Ozaki M. (2008). Local liquid crystal alignment on patterned micrograting structures photofabricated by two photon excitation direct laser writing. Appl. Phys. Lett..

[64] Ji Z., Zhang X., Shi B., Li W., Luo W., Drevensek-Olenik I., Wu Q., Xu J. (2016). Compartmentalized liquid crystal alignment induced by sparse polymer ribbons with surface relief gratings. Opt. Lett..

[65] Kitson S., Geisow A. (2002). Controllable alignment of nematic liquid crystals around microscopic posts: Stabilization of multiple states. Appl. Phys. Lett..

[66] Kitson S.C., Edwards E.G., Geisow A.D. (2008). Designing liquid crystal alignment surfaces. Appl. Phys. Lett..

[67] Ji Z., Zhang X., Zhang Y., Wang Z., Drevensek-Olenik I., Rupp R.A., Li W., Wu Q., Xu J. (2017). Electrically tunable generation of vectorial vortex beams with micro-patterned liquid crystal structures. Chin. Opt. Lett..

[68] Berreman D.W. (1973). Alignment of liquid crystals by grooved surfaces. Mol. Cryst. Liq. Cryst..

[69] Frank F.C.I. (1958). Liquid crystals. On the theory of liquid crystals. Discuss. Faraday Soc..

[70] Chen H., Zhu R., Zhu J., Wu S.-T. (2015). A simple method to measure the twist elastic constant of a nematic liquid crystal. Liq. Cryst..

[71] Li J., Wen C.H., Gauza S., Lu R., Wu S.T. (2005). Refractive Indices of Liquid Crystals for Display Applications. J. Disp. Technol..

[72] Rapini A., Papoular M. (1969). Distorsion D’une Lamelle Nématique Sous Champ Magnétique Conditions D’ancrage Aux Parois. J. Phys. Coll..

[73] Napoli G. (2006). Weak anchoring effects in electrically driven Freedericksz transitions. J. Phys. A Math. Gen..

[74] Costa Pereira A.E., Rosato A. (1975). Transmission of Nematic Liquid Crystals in Electric Fields. Rev. Bras. Fis..

[75] Gaylord T.K., Moharam M.G. (1985). Analysis and applications of optical diffraction by gratings. Proc. IEEE.

[76] Introduction to S4—S4 1.1 Documentation. https://web.stanford.edu/group/fan/S4/.

[77] Solodar A., Cerkauskaite A., Drevinskas R., Kazansky P.G., Abdulhalim I. (2018). Ultrafast laser induced nanostructured ITO for liquid crystal alignment and higher transparency electrodes. Appl. Phys. Lett..

[78] Li L. (1996). Use of Fourier series in the analysis of discontinuous periodic structures. J. Opt. Soc. Am. A.

[79] Popov E., Neviere M., Gralak B., Tayeb G. (2002). Staircase approximation validity for arbitrary-shaped gratings. J. Opt. Soc. Am. A.

[80] Mertelj A., Lisjak D. (2017). Ferromagnetic nematic liquid crystals. Liq. Cryst. Rev..

[81] Tsakonas C., Davidson A.J., Brown C.V., Mottram N.J. (2007). Multistable alignment states in nematic liquid crystal filled wells. Appl. Phys. Lett..

[82] Walton J., Mottram N.J., McKay G. (2018). Nematic liquid crystal director structures in rectangular regions. Phys. Rev. E..

[83] Majumdar A., Lewis A. (2016). Multistable nematic wells: Modelling perspectives, recent results and new directions. Liq Cryst..

[84] Migara L.K., Song J.K. (2018). Standing wave-mediated molecular reorientation and spontaneous formation of tunable, concentric defect arrays in liquid crystal cells. NPG Asia Mater..

